# Advances in Minimally Invasive Esophagectomy—An Overview of Recent Developments and a Novel Classification of Innovations in Treatment of Thoracic Esophageal Cancer

**DOI:** 10.3390/medicina61122176

**Published:** 2025-12-07

**Authors:** Florin Achim, Koji Otsuka, Takeshi Yamashita, Yutaro Asagoe, Daisuke Kurita, Adrian Constantin, Silviu Constantinoiu, Ahmed Mohssen, Cristian Rosianu, Alexandru Rotariu, Alex-Claudiu Moraru, Anthony Rasuceanu, Dragos Predescu

**Affiliations:** 1 Faculty of Medicine, Carol Davila University of Medicine and Pharmacy, 050474 Bucharest, Romania; 2 Department of General and Esophageal Surgery, Center of Excellence in Esophageal Surgery, Sf. Maria Clinical Hospital, 011192 Bucharest, Romania; 3 Division of Gastroenterological and General Surgery, Department of Surgery, School of Medicine, Showa Medical University, Tokyo 142-8666, Japan; 4 Digestive Disease Center, Showa Medical University Koto Toyosu Hospital, Toyosu 5-1-38, Koto-ku 135-8577, Japan; 5 Esophageal Cancer Center, Showa Medical University Hospital, 1-5-8 Hatanodai Shinagawa-ku, Tokyo 224-8503, Japan; 6 Department of Anesthesiology and Intensive Care Medicine, National Cancer Center Hospital, Tokyo 104-0045, Japan; 7 Department of Esophageal Surgery, National Cancer Center Hospital, Tokyo 104-0045, Japan; 8 Department of Gastroenterology, Sf. Maria Clinical Hospital, 011192 Bucharest, Romania

**Keywords:** novel classification of innovations, minimally invasive esophagectomy, McKeown modified triple approach, robotic esophagectomy, thoracic esophageal cancer, transcervical esophagectomy

## Abstract

Minimally invasive esophagectomy (MIE) has become increasingly prominent in the surgical management of esophageal cancer (EC) over the past three decades. The adoption of minimally invasive techniques has significantly enhanced oncologic esophageal surgery by improving safety, achieving oncological radicality, preserving physiological function, and elevating the postoperative quality of life of the patients. The complexity of MIE lies in its technical nuances, which critically influence postoperative morbidity and, in severe cases, mortality, especially when complications evolve unchecked. These risks underscore the importance of meticulous surgical execution and perioperative management. The optimization of mediastinal lymphadenectomy and the reduction of procedure-related morbidity have consistently represented focal points of scientific inquiry and clinical refinement, posing a persistent challenge for esophageal surgeons. MIE is widely regarded as one of the most technically demanding procedures in oncologic surgery. Its advantages, however, are most evident in the postoperative phase, where reduced trauma and faster recovery are key benefits. Experienced surgical teams have introduced refinements to MIE protocols, aiming to optimize precision and reduce complication rates. This study aims to systematically synthesize the main technological advancements and innovations currently employed in the minimally invasive management of EC, presenting them in a structured classification designed to be both accessible and practical for specialists engaged in this domain.

## 1. Introduction

Esophageal cancer ranks as the eleventh most frequently diagnosed malignancy and the seventh leading cause of cancer-related mortality worldwide [1]. For patients with resectable disease, esophagectomy combined with chemoradiotherapy remains the cornerstone of multimodality treatment. Esophagectomy with reconstruction is recognized as one of the most technically demanding procedures in the management of gastrointestinal malignancies. In the light of relatively poor overall survival, reducing the invasiveness of esophageal resection while preserving oncological radicality has become a central objective of multidisciplinary care, with the aim of improving postoperative quality of life [2].

Over the past decades, MIE has gained increasing acceptance and is now widely performed. Thoracoscopic and laparoscopic approaches have been shown to reduce perioperative morbidity and mortality in high-volume centers [3]. Evidence from large series and randomized clinical trials consistently demonstrates that MIE is associated with lower postoperative morbidity compared with open esophagectomy, without compromising oncological outcomes [4].

MIE is an advanced technical surgical procedure with a prolonged learning curve [5]. Training in MIE within specialized esophageal centers is essential for skill acquisition and procedural safety. Advanced technical skills in minimally invasive surgery and the supervision of a mentor are mandatory for the first operations. While the direct involvement of surgical trainees in complex stages of esophagectomy remains a subject of debate, it is increasingly considered feasible under structured supervision. In specialized centers, operative efficiency is enhanced by assigning the laparoscopic and neck stages to distinct assistant surgical teams. The current generation of esophageal surgeons is being trained primarily in minimally invasive and robotic techniques, often with limited exposure to open esophagectomy—a trend reminiscent of early adoption of minimally invasive techniques in digestive surgery. In contrast to other digestive surgeries, which have benefited from the complementary advancement of interventional gastroenterology, esophageal surgery lacks such procedural redundancy. Therefore, surgeons specializing in MIE must also maintain proficiency in an open approach to ensure comprehensive operative capability [6].

MIE can be performed safely after neoadjuvant treatment, which may include chemotherapy, immunotherapy, and chemoradiotherapy [7,8] nowadays. Considering the results from the immediate postoperative period, but also the long-term oncological, conventional MIE has become the standard surgical treatment in some countries, given the low availability and high cost of using robotic technology [9].

In our tertiary center, ultra-specialized in esophageal pathology, MIE by the modified McKeown triple approach was introduced in 2015. Our surgical technique using a 3D HD video camera and 4K UHD optic technology has been described previously [10]. The early outcomes of using the minimally invasive and hybrid approach in the treatment of EC (57 patients) in our department include the reduction of perioperative morbidity, the duration of hospitalization, and a faster recovery.

## 2. Objectives

This article aims to present the recent advances in the treatment of esophageal thoracic cancer through a minimally invasive approach. We will also analyze various surgical practices, some recently changed based on the experience gained in minimally invasive surgery, including surgical robots.

Certain new techniques and technologies have demonstrated favorable clinical outcomes, while others have been ineffective. Presenting these results in an objective analysis will help clinicians to decide whether or not to implement certain technologies in current practice.

## 3. Methods

This article was written following the Narrative Review Report Checklist. Two independent authors (FA, AC) searched medical databases for papers published in the last 5 years (January 2020–July 2025).

### Literature Search Strategy

**Figure 1:**
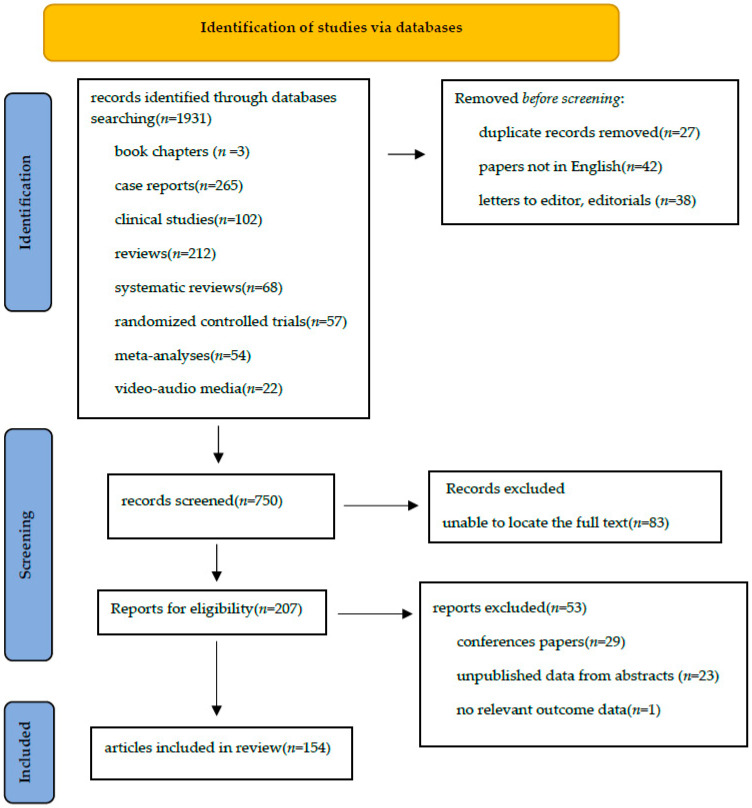
The PRISMA diagram highlights multiple studies that were assessed for eligibility. A large number of articles (553) were found dedicated in the last 5 years to the use of surgical robots in the treatment of EC.

A comprehensive literature search was conducted across PubMed, PubMed Central, EMBASE, UpToDate, Scopus, MEDLINE, and Cochrane Library, covering the period from January 2020 to July 2025. The following search terms were used: “minimally invasive esophagectomy”, “robotic esophagectomy”, “robot-assisted esophagectomy”, “thoracoscopic esophagectomy”, “thoraco-laparoscopic esophagectomy”, “transhiatal esophagectomy”, “transcervical esophagectomy”, and “thoracic esophageal cancer”. Studies were eligible for inclusion if they involved human subjects aged ≥18 years and reported technical details, operative techniques, or the use of new surgical instruments and medical devices in esophagectomy. Exclusion criteria comprised non-English publications, congress abstracts, letters to the editor, editorials, duplicate studies, and case reports. The initial search resulted in 1931 articles. After removal of duplicates and preliminary screening, 1103 titles and abstracts were reviewed by authors FA, AC, and DP. A total of 750 records were curated based on relevance to the study objectives (Figure 1).

## 4. Results

A systematic literature review identified numerous recent advances in the minimally invasive treatment of thoracic EC. Upon analysis, it was recognized that there was a need for a structured classification of these innovations, reflecting the growing interest among specialized surgical teams in developing novel techniques and integrating emerging technologies into routine practice.

Accordingly, a classification of innovations in minimally invasive treatment of thoracic esophageal cancer (Table 1) is proposed, encompassing five distinct domains: (1) technological innovations, (2) advances in surgical approach, (3) novel lymph node dissection strategies, (4) graft preparation and esophageal reconstruction, and (5) innovations in esophagogastric anastomosis.

Each category within the proposed classification includes three distinct subclasses, designed to capture the breadth of innovation in minimally invasive surgical treatment of thoracic EC. To enhance interpretability and clinical relevance, definitions and objectives have been provided for each subclass, outlining the specific role and intended impact of the respective innovations on surgical technique, perioperative outcomes, and oncologic efficacy.

### 4.1. Class 1—Technological Innovations

This category encompasses advancements that enhance the safety, precision, and efficiency of minimally invasive esophagectomy. It includes the following:

- Improvement in visualization systems: high-definition cameras, 3D imaging, and fluorescence-guided or other dye techniques that optimize the surgical field and facilitate meticulous dissection.
- Development of advanced surgical instruments: ergonomic, articulating, energy-based tools and staplers that enable complex maneuvers in confined anatomical spaces.
- Auxiliary equipment and anesthetic innovations: enhanced insufflation systems, integrated operating platforms, and anesthetic protocols tailored to thoracoscopic and robotic procedures, all contributing to intraoperative stability and reduced complication rates.

### 4.2. Subclass 1A Advances in Camera Technology

This subclass includes technological innovations in the field of optics used for intraoperative visualization, both conventionally in white light and in infrared. Current generations of cameras used in minimally invasive surgery include optimized image stabilization, detail recognition, superior autofocus, high-resolution sensors that allow detection of the depth of the operating field in low light with brightness adaptation, three-dimensional imaging, and virtual reality or artificial intelligence applications. Infrared visualization of tissues previously injected with a fluorescent substance allows for much easier and safer dissection, reducing the risk of intraoperative complications.

#### 4.2.1. Ultra-High Definition and Tridimensional Image in MIE

*Three-dimensional HD and 4K UHD.* MIE provides superior visualization for mediastinal lymphadenectomy, particularly in anatomically challenging regions in the upper mediastinum, such as the vicinity of the recurrent laryngeal nerves (RLN) and beneath the tracheal bifurcation [11]. Enhanced image quality through intraoperative video augmentation—using either 3D high definition (HD) or 4K ultra HD systems—facilitates precise delineation of dissection planes, reduces intraoperative risk, and supports comprehensive periesophageal and perigastric lymphadenectomy, thereby improving oncologic staging accuracy [12]. Comparative studies evaluating 3D HD versus 4K UHD imaging technologies have generated mixed results [13]. While some authors report reduced operative time and improved depth perception in the mediastinum with 3D systems, a substantial number of esophageal surgeons continue to utilize HD or 4K UHD, reflecting variability in institutional preference and surgeon experience [14].

**Figure 2:**
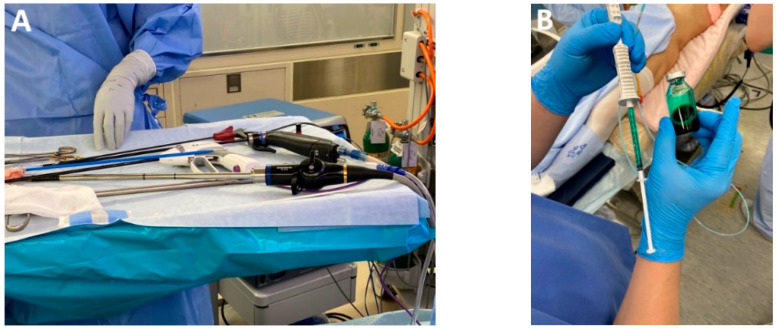

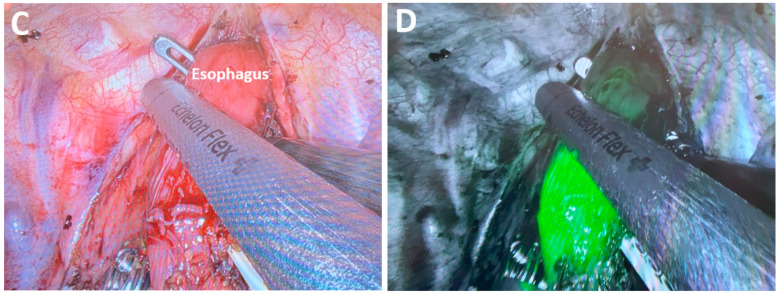
(**A**) Endoeye Flex articulating videoscope. (**B**) Endoscopic preparation for intraoperative localization of the esophageal tumor with ICG. (**C**) Intraoperative upper mediastinal view under white light. (**D**) Intraoperative near-infrared view highlighting ICG fluorescence in the upper thoracic esophagus. Endoscopic submucosal injection is performed proximal to the tumor to prevent transmural esophageal transection.

*Articulating HD 3D videoscope.* The integration of a 5 mm or 10 mm deflectable 3D HD videoscope (Endoeye Flex 3D, Olympus, with 100 degrees of articulation) into minimally invasive esophageal cancer surgery significantly enhances mediastinal dissection, particularly within the constrained anatomy of the superior thoracic aperture (Figure 2A). Its improved angulation and stereoscopic visualization facilitate precise tissue handling and safer dissection. These advantages are especially pertinent in minimally invasive transcervical esophagectomy (MICE), where the enhanced maneuverability and depth perception of the device may contribute to improved operative efficacy and reduced morbidity [15].

#### 4.2.2. Fluorescence-Guided Surgery and MIE

*Indocyanine green (ICG) for intraoperative tumor localization.* When intraoperative tumor localization is required, preoperative supratumoral injection of indocyanine green (ICG) under endoscopic guidance may be utilized. This technique is particularly advantageous during thoracoscopic or robotic esophagectomy performed in the prone position, where intraoperative endoscopic access may be limited and fluorescence guidance facilitates accurate tumor localization and resection planning, or if the transection of the upper thoracic esophagus is required (Figure 2B–D).

*Fluorescence angiography with ICG*. ICG is currently also used to assess the perfusion of the esophageal anastomoses; however, its interpretation remains largely subjective and operator-dependent [16,17]. The use of ICG has improved graft preparation techniques by enabling the identification and exclusion of poorly perfused areas, thereby reducing the risk of postoperative ischemia of the gastric conduit [18].

The implementation of ICG fluorescence imaging necessitates access to advanced endoscopic platforms equipped with infrared capabilities, as well as the routine availability of indocyanine green. These requirements support its use primarily in high-volume centers with appropriate infrastructure. Notably, reports from both peer-reviewed literature and informal clinical communications suggest that certain institutions, despite initially adopting ICG in large patient cohorts, have subsequently discontinued its use without clearly articulating the underlying rationale. Nevertheless, current evidence supports the feasibility and safety of ICG application in esophageal cancer surgery [19].

Nonetheless, concerns regarding its safety have been raised. In a retrospective study of 181 patients undergoing esophageal reconstruction (59 with ICG vs. 122 without), Bank et al. reported a significantly higher incidence of anastomotic fistula in the ICG group (10.2% vs. 1.6%, *p* = 0.015), as well as increased 90-day mortality (8.5% vs. 1.6%, *p* = 0.04). Moreover, abnormal fluorescence was strongly associated with elevated rates of anastomotic leakage (71.4% vs. 1.9%, *p* < 0.001) and 30-day mortality (28.6% vs. 0%, *p* = 0.012), underscoring the need for cautious interpretations and further validation of this technique [20].

*Tracheobronchial fluorescence (ICG-TBF) during MIE.* Upper airway administration of indocyanine green via nebulization was evaluated by Thammineedi et al. and demonstrated feasibility in a cohort of 37 patients undergoing mediastinal dissection of the esophagus from the tracheobronchial tree. Aerosolized ICG achieved a 94.6% success rate in identifying the trachea and bronchi. The technique may be particularly valuable in salvage esophagectomy, especially in patients with invaded paratracheal node involvement or tumors of the upper thoracic esophagus, where dissection from the tracheal membrane poses significant technical challenges [21].

*Near infra-red fluorescence and ICG FireFly™ for identification of the thoracic duct (TD).* TD identification during thoracoscopic esophagectomy can be achieved through ultrasound-guided peripheral injection of ICG, typically administered at the inguinal region or the bilateral first web spaces of the feet [22]. Intraoperative infrared visualization platforms, including ICG FireFly™ used in robot-assisted minimally invasive esophagectomy (RAMIE), enable real-time mapping of the thoracic duct trajectory and facilitate safe dissection in high-risk areas, such as the azygos vein and the upper thoracic esophagus above the aortic arch [23]. Barnes et al. explored alternative ICG delivery routes for TD visualization. Enteral administration via feeding jejunostomy proved ineffective, whereas intramesenteric injection successfully delineated the TD and identified ductal injury in four patients [24].

*ICG for lymphatic drainage pattern of esophageal cancer.* Fluorescence-guided detection of periesophageal and perigastric lymph nodes has been facilitated by peritumoral injection of ICG administered on the day prior to surgery. In the ESOMAP trial, Muller et al. employed intraoperative infrared imaging during RAMIE to construct a fluorescence-based map of periesophageal lymph nodes. Among 20 patients included in the study, 15 fluorescently identified nodes were resected and subsequently confirmed to be histologically negative for malignancy, suggesting a potential correlation between intraoperative fluorescence patterns and pathological outcomes [25].

#### 4.2.3. Dye-Free Imaging

*Hyperspectral imaging and real-time oximetry.* Hyperspectral imaging (HSI) enables assessment of tissue oxygenation by analyzing the spectral reflectance properties of the gastric conduit and esophageal stump. This technology may assist surgeons in selecting the optimal site for esophagogastric anastomosis, thereby reducing the risk of anastomotic leakage. Ilgen et al. investigated the intraoperative feasibility of laparoscopic HSI using the TIVITA® Mini System (HSI-MIS) during MIE. Perfusion measurements were obtained at both the proximal esophageal stump and the gastric conduit, with postoperative outcomes correlating with intraoperative findings. Despite promising results, randomized clinical trials are warranted to validate the utility of HSI and support its routine intraoperative application [26].

An alternative approach to assessing gastric graft perfusion without the use of injectable dyes is the ELUXEO Vision System (FUJIFILM Healthcare Americas Corporation, Lexington, MA), which enables real-time monitoring of tissue O_2_ saturation through oxygen saturation endoscopic imaging (OXEI). This technique has demonstrated feasibility and offers distinct advantages in patients with contraindications to ICG, allowing for continuous intraoperative assessment and facilitating timely surgical decision-making [27]. Its safety and overall simplicity, as observed in patients enrolled in randomized clinical trials, support the potential for broader validation and future integration into routine surgical practice.

#### 4.2.4. Artificial Intelligence

*Intraoperative Image AI analysis and MIE.* Artificial intelligence (AI) has demonstrated potential in accurately identifying anatomical structures and enhancing the safety of complex mediastinal dissections, particularly in cases involving lymph node invasion adjacent to the RLN or intrathoracic organs [28]. The Surgomics platform applies machine learning algorithms to intraoperative data collected during MIE to generate personalized predictions of procedural risks and postoperative outcomes [29]. Additionally, Surgomics can be leveraged to objectively assess surgical proficiency and facilitate comparative analysis of operative performance across individual surgeons and institutions.

*ICG fluorescence (Stryker) and SPY-Q.* The integration of artificial intelligence into near-infrared imaging with ICG enhances the objectivity of intraoperative perfusion assessment. Software platforms such as SPY-Q enable real-time analysis of fluorescence intensity curves, allowing differentiation between true tissue perfusion and raw signal intensity. This approach supports accurate interpretation of ICG imaging and may prove intraoperative decision-making regarding graft viability and anastomotic site selection [30].

### 4.3. Subclass 1B Surgical Instruments

This subclass comprises surgical instruments designed to facilitate the execution of fine operative maneuvers, ensure the safety of essential surgical stages, and contribute to reducing the overall duration of the procedure.

#### 4.3.1. Ultra-Specialized Surgical Instruments for MIE

**Figure 3:**
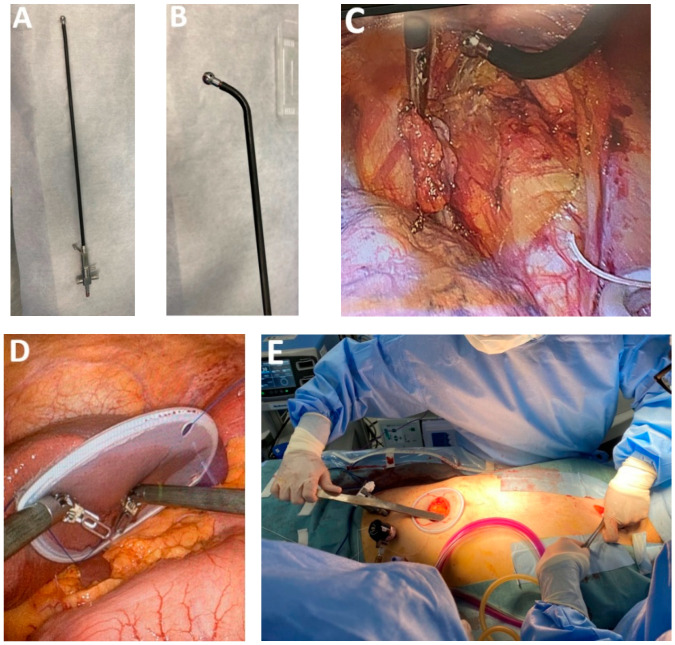
(**A**,**B**) Atraumatic suction device (Showa type) utilized for gentle tissue handling. (**C**) Articulating suction instrument and a fine-tip grasper (Murakami type) employed for precise lymph node dissection. (**D**) Silicone disc used for liver retraction (**E**) Metal spatula applied for retrosternal tunnel creation.

*Innovative surgical instruments.* Certain surgical teams devote their professional careers to the continuous refinement of esophagectomy techniques, leading to the development of patented innovative tools tailored to distinct phases of the operation. Among these, the atraumatic suction with bendable shaft (Murakami suction, Showa type, Heiwa Medical Instruments, Hofu, Japan) has demonstrated noble versatility, contributing to mediastinal dissection by gently mobilizing adjacent organs while simultaneously functioning as suction and irrigation (Figure 3A–C).

The recent introduction of the silicone disc (Hakko Medical, Chikuma, Japan), which consists of a silicone rubber membrane inside a flexible ring, has facilitated atraumatic retraction of the lung during the thoracoscopic stage and the left liver lobe during the laparoscopic stage, thereby minimizing organ injury and improving operative ergonomics (Figure 3D). Additionally, the use of fine graspers enables precise manipulations of lymphatic tissue, particularly during dissection in proximity to the RLN (Murakami lymph node grasping forceps). A specialized instrument developed to facilitate safe creation of the retrosternal tunnel is the metal spatula (Figure 3E). Its design allows controlled dissection along the posterior aspect of the sternum, minimizing the risk of injury to adjacent structures. Single-port instrumentation has also emerged as a valuable adjunct in minimally invasive approaches to EC resection.

**Figure 4:**
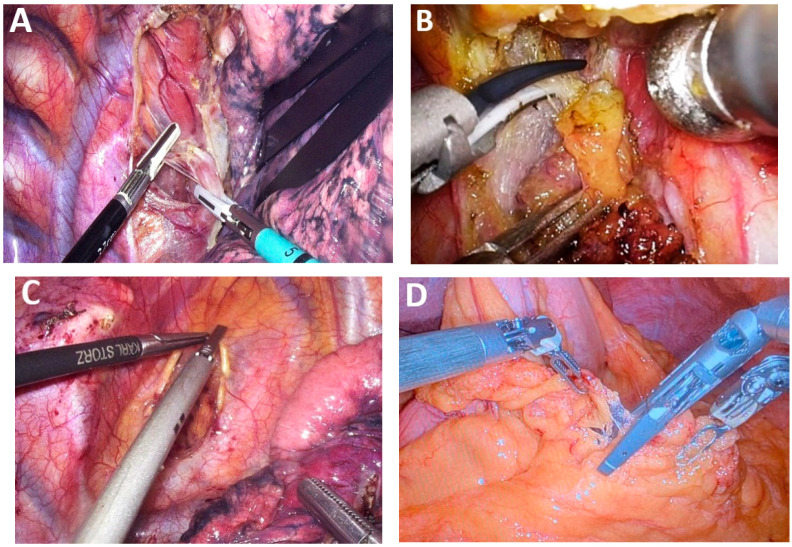
(**A**) Division of aorto-esophageal arteries using the Ligasure device. (**B**) Lymphadenectomy of station 106 recL performed with the Harmonic scalpel. (**C**) Incision of uppermediastinal pleura with Sonicision. (**D**) Application of Da Vinci Vessel Sealer during abdominal phase of the procedure.

*Hemostasis instruments.* LigaSure™ Maryland Laparoscopic Sealer (Medtronic, Galway, Ireland) and Harmonic™ (Ethicon, Johnson&Johnson Med Tech, New Brunswick, New Jersey, USA) remain the most widely utilized instruments for dissection and hemostasis in minimally invasive esophageal cancer surgery (Figure 4A,B). Additional instruments employed for hemostasis in MIE include Da Vinci Vessel Sealer (Intuitive, Sunnyvale, California, USA), Sonicision™ (Medtronic, Galway, Ireland) and Thunderbeat™ with Intelligent Tissue Monitoring (ITM) (Olympus, Hachioji, Tokyo, Japan), which integrate advanced safety technology to enhance dissection precision and vascular control (Figure 4C,D).

*Bipolar vessel sealing* vs. *ultrasonic energy devices in thermal damage of laryngeal nerve.* Bipolar energy devices generate heat through direct electrical contact with tissue, resulting in elevated peak temperatures, while ultrasonic instruments produce frictional heat via high-frequency mechanical vibrations. Both modalities carry a risk of collateral thermal injury to adjacent structures during dissection. Multiple studies have compared bipolar vessel sealing and ultrasonic energy devices in the context of mediastinal dissection and lymphadenectomy along the RLN, achieving inconsistent results [31,32]. Bipolar vessel sealers have demonstrated superior protection against inadvertent thermal damage to critical mediastinal structures in some reports [33,34]. Ultimately, the choice of instrument depends on the surgeon’s preference, experience, and availability, particularly when operating in proximity to the RLN.

#### 4.3.2. Advances in Endoscopic Stapling

*Linear and Circular Powered Stapler (ECP) with Gripping Surface and Tri-Staple™ Technology (GST) and 3D staples*.

**Figure 5:**
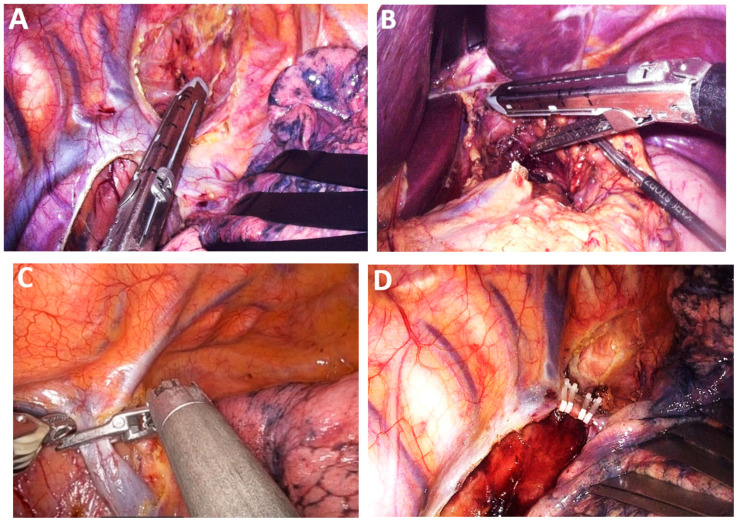
(**A**) Division of the azygos vein with a vascular stapler. (**B**) Transection of left gastric artery with a vascular stapler. (**C**) Robotic-assisted clipping of the azygos vein with Hem-o-lok polymer clips. (**D**) Clipping the azygos vein confluence with four Hem-o-lok clips.

Anastomotic healing is key to postoperative recovery in patients undergoing surgery for EC. Inadequate healing, particularly the development of anastomotic leakage, poses significant septic risks that may become life-threatening if not promptly identified and managed. To enhance anastomotic integrity, mechanical stapling devices—both linear and circular—have been introduced alongside conventional hand-sewn techniques. These devices incorporate advanced technologies, including automated firing and tissue approximation with adjustable height and tridimensional staple configurations, aiming to standardize esophagogastric anastomosis and reduce technical variability [35]. The addition of multiple staple rows has further minimized the need for supplementary reinforcement, streamlining the procedure while maintaining structural reliability [36]. During the vascular stages of esophagectomy, substitution of standard vascular stapling cartridges with self-locking polymer clips (Hem-o-lok) proved feasible and cost-effective with compatibility across platforms, including surgical robots (Figure 5A–D).

### 4.4. Subclass 1C Auxiliary Equipment

This subclass includes advanced auxiliary equipment, including modern real-time smoke evacuation systems and other specialized devices that support the precise execution of delicate surgical maneuvers.

*Smoke evacuation systems and MIE.* Insufflation systems equipped with continuous smoke evacuation, such as AirSeal® (ConMed, Utica, NY, USA), provide stable CO_2_ pressure and enhanced intraoperative visibility—key factors during the technically demanding stages of esophagectomy. In a comparative study by Otsuka et al., patients undergoing surgery with the AirSeal® system experienced significantly shorter operative times and reduced intraoperative blood loss compared with those treated using conventional insufflators [37]. In a separate analysis, the same authors reported no significant differences in long-term oncologic outcomes between patients who received artificial pneumothorax with CO_2_ and those who did not [38].

**Figure 6:**
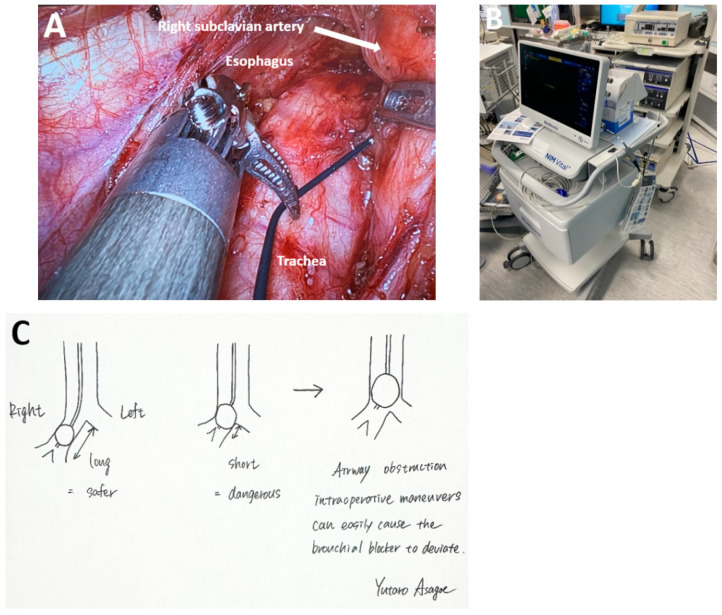
(**A**,**B**) Intraoperative neuromonitoring of the right RLN using the NIM Vital™ system (Medtronic) (**C**) Following placement of the endobronchial blocker in the right main bronchus, the distance from the tracheal bifurcation to the first bronchial branch is measured to confirm appropriate positioning. The surgical team is promptly notified of the blocker’s introduction. Given the potential for intraoperative displacement, surgeons are advised to exercise heightened caution during tracheal mobilization. In the event of blocker migration in the trachea, immediate reversion to bilateral lung ventilation is required to maintain adequate oxygenation and procedural safety. This illustration was written by Dr. Yutaro Asagoe, an anesthesiologist in the operating room of the National Cancer Center Hospital, Tokyo, Japan, in response to a query regarding airway management during MIE.

*Intraoperative monitoring of laryngeal recurrent nerve.* Intraoperative neuromonitoring of the RLN with the NIM Vital™ system (Medtronic, Galway, Ireland) is employed in cases where nerve identification is challenging or when excision of a metastatic lymph node adjacent to the nerve is required (Figure 6A,B). During dissection, the lymphofatty tissue surrounding the RLN can be meticulously resected to avoid iatrogenic injury [39,40].

*Lung separation during MIE.* The role of the anesthesia team in facilitating optimal surgical conditions during MIE is indispensable. The standard method for lung separations is the use of a double-lumen Carlens rigid catheter (Teleflex, Wayne, PA, USA), which may hinder adequate exposure for dissection along the left RLN. Endobronchial blockers (PHYCON TCB Endotracheal Tube, Uniblocker 3.0 mm (9 FR), Length 510 mm, Fuji Systems Corp., Tokyo, Japan) are preferred for selective intubation by experienced anesthesia teams.

Use of the endobronchial blocker requires an anesthesiologist skilled in bronchoscopy, as periodic verification of blocker positioning within the right main bronchus is essential to prevent proximal migration into the trachea, which can compromise ventilation of the left lung. To facilitate prompt recognition and correction of bronchial blocker displacement, anesthesiologists must maintain situational awareness of the surgical workflow and actively monitor the operative field (Figure 6C). Prior to procedures with an elevated risk of blocker migration, the fraction of inspired oxygen (FiO_2_) should be set to 1.0 to ensure adequate oxygenation and allow sufficient time for safe repositioning if displacement occurs. Dedicated training of the anesthesia personnel in esophageal resection protocols is critical to minimizing intraoperative anesthetic-surgical incidents and optimizing postoperative outcomes.

### 4.5. Class 2—Advances in Surgical Approach

The second category of innovations encompasses advances in the surgical approach of MIE, RAMIE, and transcervical esophagectomy. These developments aim to reduce surgical trauma, improve access to anatomically challenging regions, and refine operative technique. Collectively, they contribute to minimizing intraoperative complications and shortening the procedural learning curve.

### 4.6. Subclass 2A Transthoracic MIE

This subclass comprises approaches specifically designed to reduce the morbidity associated with conventional thoraco-laparoscopic esophagectomy. It also includes refined surgical techniques that facilitate complex maneuvers during mediastinal dissection of the esophagus and laparoscopic dissection of the future graft.

**Figure 7:**
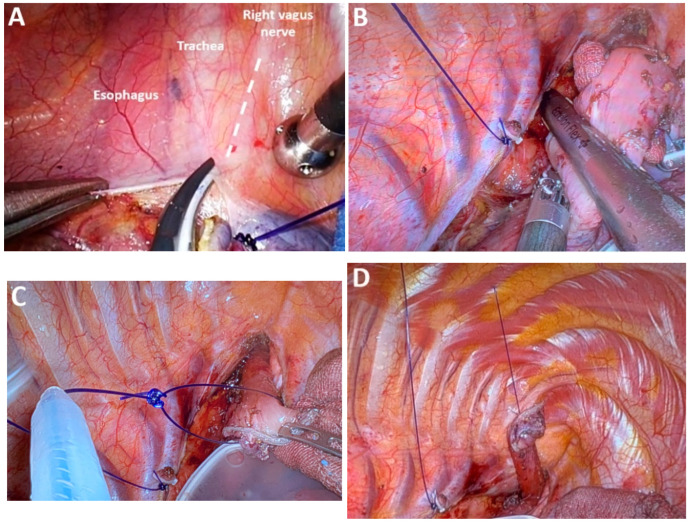
(**A**) Right cranial nerve guides cranial dissection of upper mediastinum. (**B**) Traction applied to the azygos vein stump to aid exposure and transection of the esophagus during thoracic phase of the procedure. (**C**) Intraoperative view following transection and anchoring of the esophagus. (**D**) Enhanced superior mediastinal dissection facilitated by anchoring the azygos vein stump and proximal esophagus.

*Dissection of the upper thoracic esophagus*. The potential advantages of prone and semi-prone positioning over the traditional left lateral decubitus approach during thoracoscopic esophageal dissection have been discussed within the surgical community in the last 10 years. The thoracoscopic stage consists of multiple steps, the sequence of which may vary depending on the surgical team’s experience, mediastinal anatomy, and the presence of pleural adhesions. Surgeons may employ either Thoracoport-type trocars or trocars equipped with CO_2_ insufflation systems (artificial pneumothorax at 8 mmHg), selected according to the operative strategy and technical demands. With the right lung collapsed, dissection begins with divisions of the upper mediastinal pleura above the azygos vein, providing access to the right paratracheal lymph nodes adjacent to the right RLN (station No. 106recR, Japanese Classification of Esophageal Cancer, 12th Edition) [41,42]. The right vagus nerve serves as a reliable anatomical landmark, guiding cranial dissection and facilitating identification of the recurrent nerve as it loops beneath the right subclavian artery (Figure 7A). Dissection of the upper thoracic esophagus may be limited to the level of the inferior thyroid artery; however, in certain cases, the dissection may need to be extended more cranially.

*Traction on azygos vein stump*. One of the key vascular steps during esophagectomy involves division of the azygos vein arch, which facilitates access to the mediastinum for circumferential dissection of the esophagus and adjacent lymphatic tissue. Following transection of the azygos vein—either with a vascular stapler or via Hem-o-lok clipping—the residual vein stumps may be secured using Endoloops. Traction can then be applied through externalized sutures to enhance operative exposure and optimize visualization of the surgical field (Figure 7B).

*Sectioning the upper thoracic esophagus.* An alternative strategy to enhance visualization within the mediastinal surgical field involves transecting the upper thoracic esophagus above the level of the azygos vein arch (Figure 7B). This approach avoids division of the right bronchial vessels and the azygos vein itself. The resulting esophageal stump is easily maneuverable and can be anchored using a transcutaneous suture externalized at the upper chest wall (Figure 7C,D). This facilitates dissection of the upper thoracic inlet, particularly around the left RLN (group 106recL, Japanese Classification of Esophageal Cancer, 12th Edition).

#### Reducing the Risk of RLN Damage

**Figure 8:**
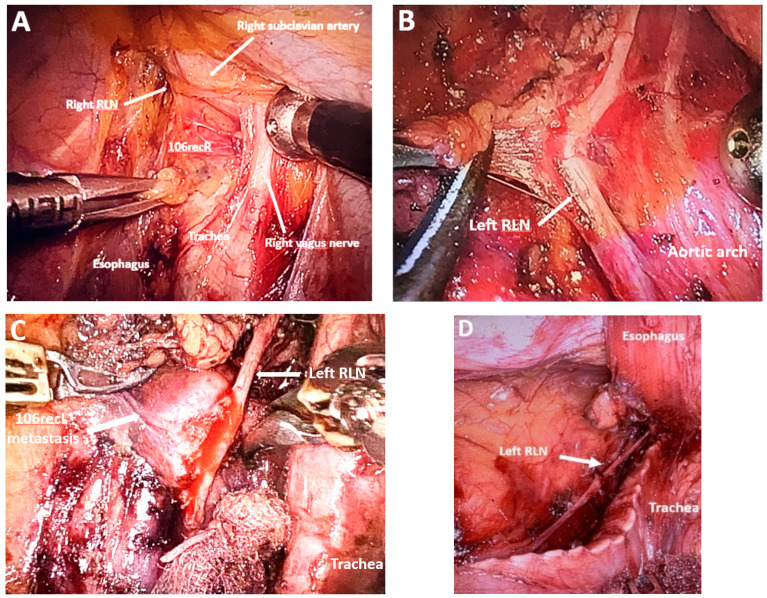
(**A**) Lymphadenectomy of station 106recR (rightRLN). (**B**) Lymphadenectomy of station 106recL (left RLN). (**C**) Lymphadenectomy 106recLeft with resection of metastasis. (**D**) Intraoperative view following 106recLeft lymphadenectomy. The dissection of the lymph nodes surrounding the left RLN (106recL) is significantly facilitated by improved tracheal mobility, a condition achievable only through the use of an endobronchial blocker.

*“Native tissue preservation” technique.* Otsuka et al. have proposed a technique refined through extensive experience in the minimally invasive management of locally advanced EC, aimed at reducing the risk of RLN injury (Figure 8A,B). Termed the „*native preservation technique*”, this approach utilizes preserved connective tissue as a dissection landmark near the recurrent nerves, thereby minimizing the risk of nerve damage from direct traction (Figure 8C,D) [43].

*“Elastic suspension of left RLN” technique.* To minimize the risk of left RLN injury, Donming et al. employed a technique involving suspension of the nerve using an elastic traction strap. This approach facilitated safer manipulation during lymphadenectomy of the 106recL nodal station [44].

*En bloc upper mediastinum lymph node resection.* Zhu et al. introduced an innovative en bloc lymphadenectomy technique targeting lymph nodes adjacent to the left RLN. This approach resulted in a higher yield of resected lymph nodes and a reduced incidence of nerve injury; however, these differences did not reach statistical significance [45].

*Outermost layer-oriented approach in mediastinal dissection.* To reduce the risk of RLN injury while maintaining oncologic rigor during lymphadenectomy, Nakauchi et al. proposed an outermost layer-oriented dissection technique around the RLN, combined with intraoperative nerve monitoring (IONM)—a method adapted from gastric cancer surgery. Short-term outcomes associated with this approach were favorable [46].

*Comparison of lymph node resection techniques.* Wang et al. conducted a comparative analysis of two lymphadenectomy techniques along the left RLN: a retraction and a suspension method. The retraction technique significantly reduced operative time (*p* < 0.001), while postoperative outcomes were extensively analyzed and found to be comparable between the two approaches [47].

*Recurrent nerve size and postoperative complications*. Lye et al. conducted a retrospective study involving 149 consecutive patients who underwent RAMIE for EC. The intraoperative diameter of the recurrent laryngeal nerve was assessed using 1.5 mm as a reference threshold, and it was found that thinner left RLNs were strongly associated with increased risk of permanent postoperative RLN palsy. Based on these findings, they recommend that surgeons proactively assess nerve diameter and adjust their technique accordingly to minimize the risk of nerve injury [48].

*Bilateral pleural drainage through the opening of the left pleura.* At the end of the thoracoscopic stage, an incision in the left pleura may be performed at the level of the lower mediastinum to facilitate placement of a drain for evacuating both pleural cavities. Practices regarding the type of drainage system and the number of chest tubes vary among surgical teams, reflecting differences in institutional protocols and individual surgeon preferences. Opening the left pleural cavity remains a subject of debate, with both supporting and opposing viewpoints. The primary concern against this approach is the potential increase in procedural morbidity due to the creation of an additional surgical field. However, several arguments support left pleural drainage. One rationale for the need to drain the left pleura is that the left lung often sustains ventilatory trauma during esophagectomy due to prolonged high pressure of one-lung ventilation and will develop postoperative atelectasis and pleurisy. Another advantage is the opportunity to achieve more through resection of lymphofatty tissue from the middle and lower mediastinum. The decision to employ a postoperative aspiration thoracic drainage using conventional chest drainage systems or digital chest drainage systems is influenced by the surgical team’s experience and by the choice of graft ascent to the cervical region.

**Figure 9:**
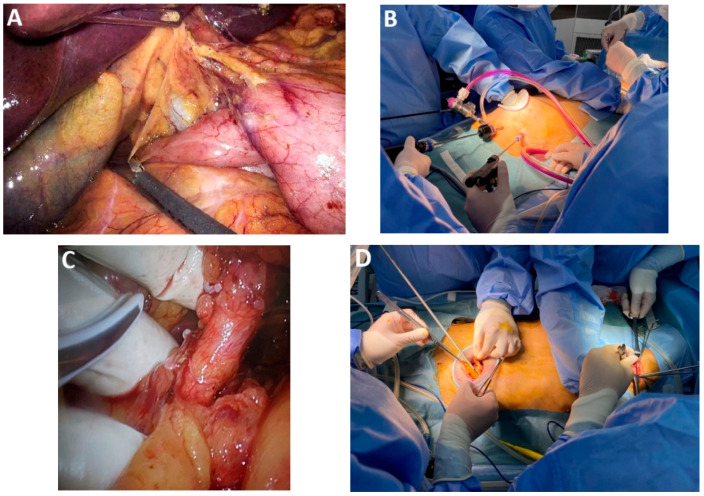
(**A**) Duodenopancreatic detachment. (**B**) Hand-assisted laparoscopic approach during abdominal phase. (**C**) Dissection of the left gastric artery. (**D**) Concurrent abdominal and cervical dissection.

*MIE—Laparoscopic and neck stage*. During the laparoscopic and neck stage, the patient is positioned supine, and five trocars are typically utilized. Laparoscopic mobilization of the stomach carries a risk of bleeding or injury to the left gastroepiploic pedicle and short gastric vessels, particularly during the early stages of the learning curve. Key factors in preventing graft ischemia include careful evaluation of venous drainage, appropriate sizing of the hiatal opening, and the tension-free mobilization achieved through duodeno-pancreatic detachment of extracorporeal preparation (Figure 9A). Calibration of the hiatal aperture and fixation of the graft to the hiatus are necessary in preventing diaphragmatic hernia, especially given the reduced intraperitoneal adhesions associated with minimally invasive techniques.

*Hand-assisted laparoscopy.* Hand-assisted laparoscopy may be employed to minimize trauma to the gastric conduit caused by laparoscopic instruments (Figure 9B,C). This technique involves a 7 cm transverse incision along the midline, positioned 4 cm below the xyphoid and slightly offset to the left [49]. The transverse incision facilitates hand-assisted mobilization of the stomach and facilitates extracorporeal preparation of the gastric graft, helping to reduce risks associated with the learning curve in MIE and contributing to a reduction in operative time (Figure 9D).

*Reduced port MIE*. Enhanced access with minimal tissue trauma remains a central objective in the surgical management of EC [50,51]. Huang et al. assessed the feasibility of reducing the number of trocars and reported a shorter operative time and decreased postoperative analgesic requirements, without compromising oncologic outcomes [52].

*Single-port MIE.* Xiao et al. compared single-port MIE with the multiport approach and reported a lower incidence of mild postoperative complications, reduced postoperative pain, and shorter hospital stays [53]. Building on these findings, Weng et al. highlighted the improved postoperative aesthetic outcomes associated with single-port MIE, noting that both immediate and long-term clinical results remained unaffected [54]. Tang et al. utilized a periumbilical incision along with a single auxiliary trocar during laparoscopic surgery, demonstrating comparable outcomes to those achieved with the conventional multiport technique [55].

### 4.7. Subclass 2B: Transthoracic Robotic-Assisted Esophagectomy

This subclass incorporates surgical techniques utilizing robotic-assisted approaches. RAMIE was performed with 3D HD visualization and ICG technology. Such as the DaVinci Xi Firefly system—it offers several advantages, including safe and precise mediastinal dissection of nodal stations 106recR and 106recL, reduced risk of RLN injury, and ergonomic benefits for the surgical team [56,57]. However, robotic surgery also presents limitations, such as instrument collision within the thoracic cavity [58].

**Figure 10:**
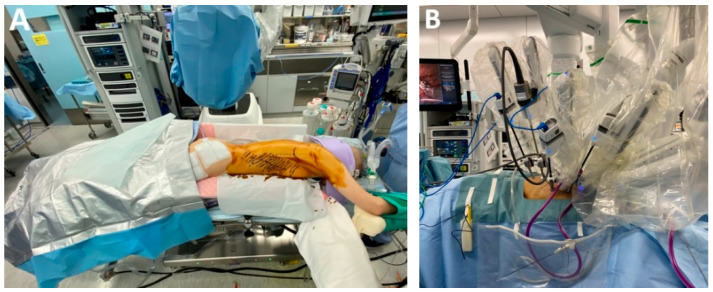

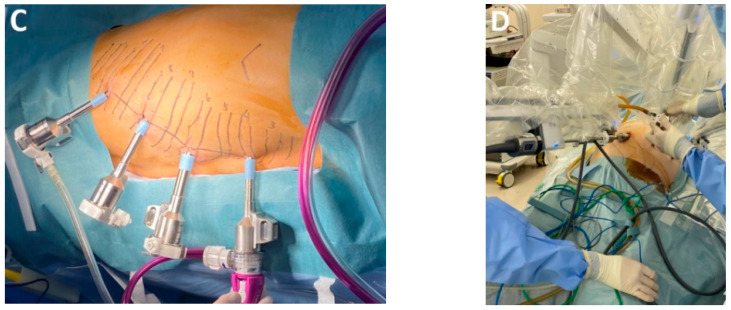
(**A**) Semi-prone position. (**B**) Position of the robotic arms—thoracic stage. (**C**) Placement of trocars—RAMIE—thoracic stage. The image illustrates the numbering of the intercostal spaces. (**D**) Robotic esophagectomy—abdominal stage.

*RAMIE left lateral decubitus* vs. *prone position.* The thoracic stage of RAMIE is typically performed with the patient in the prone position, primarily to minimize intraoperative instrument collisions (Figure 10A). The positioning also alters the approach to the mediastinal dissection compared with the left lateral decubitus position. Nonetheless, RAMIE in left lateral decubitus remains feasible, offering visibility comparable to conventional thoracoscopy while enabling rapid conversion without the need for patient repositioning (Figure 10B–D). Perioperative outcomes are also comparable to those achieved with conventional MIE [59].

*Challenges in robotic esophagectomy.* Complex thoracic anatomy can pose significant technical challenges during RAMIE, potentially compromising oncologic efficacy and procedural safety. Anatomical variations such as a narrow mediastinum or a left-shifted thoracic esophagus have been associated with prolonged operative time, reduced lymph node yield, and a higher incidence of RLN injury [60].

*Outside the Cage (OTC) Non-Intercostal RAMIE.* OTC RAMIE is an innovative approach to thoracic esophagectomy that avoids intercostal incisions. By eliminating direct trauma to the intercostal nerves, this technique aims to reduce postoperative pain and minimize complications associated with nerve injury [61].

*New docking methods.* To address the prolonged operative time often associated with robotic-assisted procedures, Sato et al. proposed a novel docking strategy for the abdominal stage of RAMIE. This revised configuration of the robotic arms enables simultaneous cervical dissection and lymphadenectomy, enhancing procedural efficiency [62].

*New robotic platforms*. Recent advancements have enhanced earlier generations of surgical robots, while newer, more cost-effective modular systems have emerged. These innovations aim to reduce intraoperative instrument conflicts and improve overall procedural efficiency [63,64]. The adoption of next-generation robotic platforms may also depend on the parallel development of specialized accessories, including advanced robotic hemostasis instruments. These adjuncts are essential for maintaining procedural safety and expanding the versatility of robotic systems in complex surgical environments.

*Robotic single-port esophagectomy*. Instrument conflict during RAMIE may be reduced by the development of next-generation robotic platforms featuring a single arm capable of accommodating multiple laparoscopic instruments. These systems incorporate specialized design features, including tailored instrument shape and working angles, to enhance maneuverability and reduce intraoperative interference [65].

*Tele-robot-assisted MIE.* The integration of mechanized systems between the operating surgeon and the patient enables remote surgical intervention [66]. By leveraging contemporary telecommunications technologies, remote MIE offers substantial clinical and educational advantages. Patients with anatomically complex or high-risk esophageal cancer may benefit from access to the expertise of high-volume specialized centers. Simultaneously, the approach facilitates telementoring for surgeons in training, promoting skill acquisition and procedural standardization [67].

*Autonomous Robotic Esophagectomy.* The competitive landscape among laparoscopic instrument manufacturers is rapidly evolving toward the development of next-generation robotic platforms. These systems represent a transitional phase toward full automation, where surgical tasks may be executed autonomously. With the integration of artificial intelligence capable of analyzing clinical parameters and selecting the optimal surgical approach, the surgeon’s role may shift predominantly to procedural oversight [68,69].

### 4.8. Subclass 2C Transcervical Approach

This subclass focuses on the transcervical approach, which, unlike the blind dissection technique described by Orringer, is conducted under direct visual guidance. This method facilitates safe and effective periesophageal lymphadenectomy.

*Origin of the transcervical approach.* The transhiatal approach avoids transpleural access, thereby minimizing the risk of respiratory complications. Indications for transhiatal MIE mirror those of the open technique and include patients with significant pulmonary or cardiac comorbidities who are unable to tolerate single-lung ventilation. In cases where dissection of the upper thoracic esophagus proves challenging, a mediastinoscope may be employed to facilitate the cervical approach [70,71].

*Transhiatal MIE with a flexible mediastinoscope.* Wu et al. introduced the use of a flexible mediastinoscope as an alternative to the conventional rigid instrument to enhance access and reduce the technical complexity associated with dissection of the upper mediastinum. Compared with the rigid mediastinoscope, the flexible approach demonstrated greater feasibility, procedural efficiency, and speed, along with an improved node yield [72].

*Minimally invasive transcervical esophagectomy (MICE).* The combined transcervical and transhiatal approach represents an MIE technique that avoids entry into the right pleural cavity. Its adoption has increased in recent years, particularly in selected patient cohorts [73]. Despite its advantages, the transcervical route may be associated with a higher incidence of RLN palsy [74]. However, MICE may pose technical challenges due to patient-specific anatomical variations in the neck and upper mediastinum. Furuke et al. identified several factors associated with increased procedural difficulty, including a steep cervical angle (*p* = 0.023), reduced distance between the vertebral bodies and the aorta (*p* = 0.002), and midthoracic esophageal tumors (*p* = 0.040) [75]. The mediastinoscopic approach typically employs single-port instruments, and a key step during the cervical phase involves identification and marking of the RLN [76,77]. The integration of EndoEye Flex 3D technology (Olympus, Hachioji, Tokyo, Japan) has further enhanced visualization and operative precision.

Vercoulen et al. implemented the transcervical esophagectomy technique in a Western tertiary center, treating 75 patients with esophageal cancer (cT1b-4aN0-3M0). The procedure achieved a 96% R0 resection rate, with a mean of 29 lymph nodes harvested and a pulmonary complication rate of 16%. RLN palsy occurred in 33 of 75 patients, with spontaneous recovery documented in 30 cases [78].

**Figure 11:**
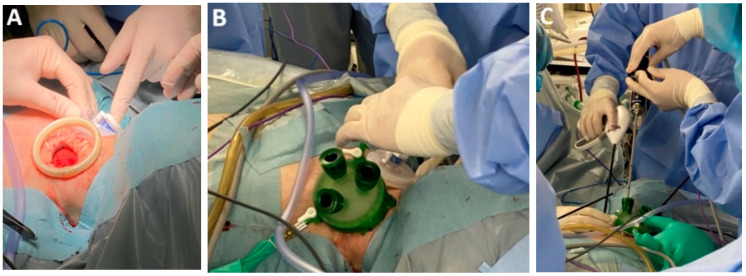

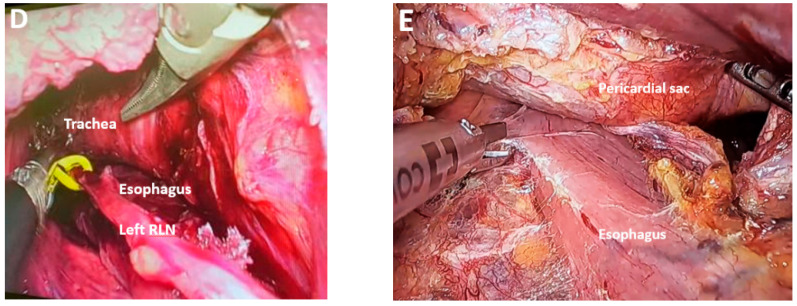
(**A**) Bilateral cervicotomy. (**B**) Preparations for transcervical approach using single-port instruments. (**C**) Transcervical approach. (**D**) Intraoperative view of mediastinal dissection surrounding the esophagus with isolation of left RLN. (**E**) Transhiatal dissection of the lower esophagus.

*Bilateral transcervical mediastinoscope-assisted transhiatal laparoscopic esophagectomy (BTC-MATLE).* The bilateral transcervical approach enhances access to the superior mediastinum and reduces instrument interference within the confined operative field (Figure 11A–E) [79].

*Robot-Assisted Transcervical Esophagectomy (RATME).* Given the anatomical constraints of the upper mediastinum and the proximity to major vascular structures, a transcervical robotic approach was used to ensure precise dissection and optimal visualization. This technique facilitates safe maneuvering in a narrow operative field and enhances exposure of vital mediastinal organs [80,81]. Daiko et al. used robotic transcervical esophagectomy via a bilateral cervical approach for a safer upper mediastinal dissection and to reduce the rate of RLN injury [82].

### 4.9. Class 3A—Lymph Node Dissection

The third class consists of novel techniques for lymph node dissection that enhance the radicality of lymphadenectomy while minimizing procedure-related complications.

### 4.10. Subclass 3A Mediastinal Lymphadenectomy

This subclass includes innovations in mediastinal lymphadenectomy techniques used in the treatment of EC.

**Figure 12:**
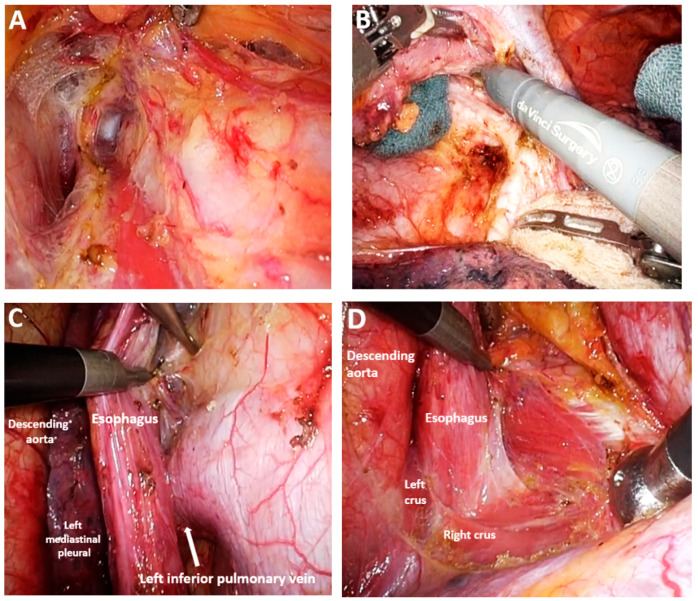
(**A**) Intraoperative view of the upper mediastinum following dissection. (**B**) Subcarinal view following lymph node dissection. (**C**) Inferior left pulmonary vein following mediastinal lymphadenectomy. (**D**) Lower mediastinum following completion of lymphadenectomy.

*Standardization of mediastinal lymphadenectomy*. Owing to divergent anatomical interpretations, the classification of mediastinal lymph nodes into distinct nodal groups remains inconsistent, despite the use of a shared terminology in describing lymphadenectomy techniques. In this study, the Japanese Classification of Esophageal Cancer, 12th Edition, was adopted to delineate mediastinal lymph node groups [41,42]. During upper mediastinal dissection, identification and preservation of the thoracic duct is important; it is typically visualized superior to the aortic arch (Figure 12A). Dissection of the middle mediastinum inferior to the aortic arch allows for identification and preservation of the left bronchial artery while enabling resection of the subcarinal lymph nodes (No. 107), right main bronchus nodes (No. 109R), left main bronchus nodes (No. 109L), and tracheobronchial lymph nodes (No. 106tbL) (Figure 12B,C).

Lower mediastinal dissection begins with the division of the inferior pulmonary ligament and the mobilization of the lower mediastinal pleura up to the middle mediastinum. This step enables resection of the middle thoracic paraesophageal lymph nodes (No. 108), lower thoracic paraesophageal lymph nodes (No. 110), pulmonary ligament nodes (No. 112pulR), anterior thoracic para-aortic nodes (No. 112aoA), posterior thoracic para-aortic nodes (No. 112aoP), and supradiaphragmatic lymph nodes (No. 111) (Figure 12D).

Thoracoscopic mediastinal dissection can be technically difficult due to anatomical, clinicopathological factors (tumor stage), or post-radiochemotherapy adhesions [83]. Intraoperative management of patients undergoing mediastinal dissection can also be difficult due to a variety of anesthetic issues (hypotension, hypoxemia, tachycardia). Communication between the surgical and anesthetic teams is essential to prevent any incidents.

*Radical mediastinal lymphadenectomy.* Radical mediastinal lymphadenectomy is associated with an increased risk of postoperative complications. Moreover, ultraradical lymphadenectomy does not guarantee prevention of local disease recurrence, and local reintervention for metastatic excision is typically not feasible, necessitating transition to palliative care.

The extent of lymph node resection in the upper mediastinum (>4 lymph nodes) is essential for accurate staging and has prognostic value for long-term oncologic outcomes in patients with upper and middle thoracic esophageal cancers [84]. However, in cases of lower esophageal tumors, the number of resected nodes in the superior mediastinum does not independently predict long-term survival [85].

*Thoracic duct resection.* Esophagectomy with radical mediastinal lymphadenectomy may include TD resection; however, its oncologic benefit remains controversial. In a study by Yu et al., TD metastases were identified in 11% of patients undergoing resection, yet the procedure did not confer a survival advantage and was not recommended for routine use, except in cases of locally advanced tumors [86]. Matsuda et al., analyzing data from the JCOG1109 clinical trial, reported that TD resection in patients undergoing esophagectomy with radical lymphadenectomy and neoadjuvant therapy did not significantly impact overall survival. Notably, a subgroup receiving neoadjuvant DCF (docetaxel, cisplatin, and 5-fluorouracil) demonstrated improved survival outcomes [87].

**Figure 13:**
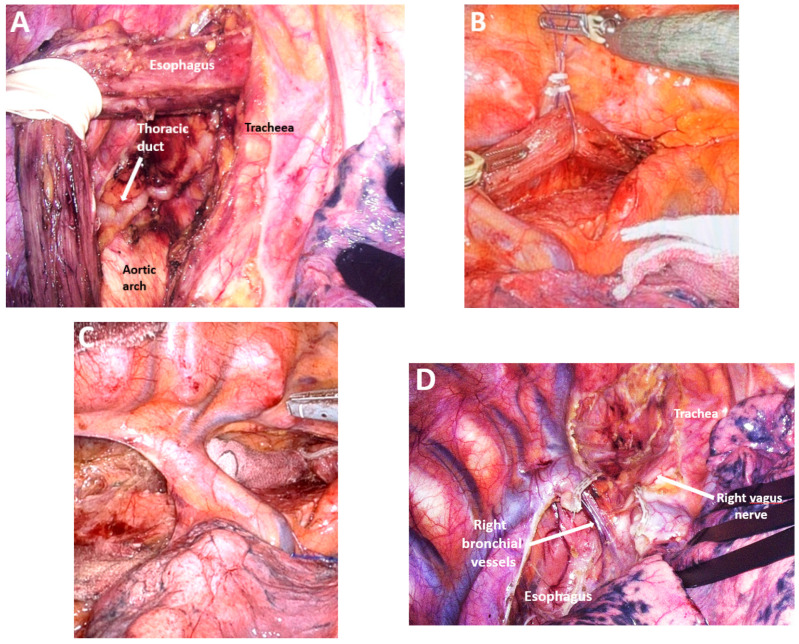
(**A**) Identification of the preserved thoracic duct located above the aortic cross. (**B**) Isolation of esophagus above the cross of the azygos vein. (**C**) Dissection of the thoracic esophagus performed to the left of the preserved cross of the azygos vein. (**D**) Mediastinal dissection of the esophagus can be performed while preserving the right bronchial vessels.

In a comparative study involving 684 patients, Igaue et al. found no significant differences in operative time, intraoperative blood loss, lymph node yield, R0 resection rate, 5-year overall survival, or recurrence-free survival between patients with and without TD resection. However, those who underwent resection exhibited higher rates of chylothorax and 90-day mortality. Although these differences were not statistically significant, routine TD resection is not recommended in patients with cT1-3 tumors who received neoadjuvant therapy (Figure 13A) [88].

#### Advances in Mediastinal Lymphadenectomy

*Two-rope method.* Wang et al. introduced a novel mediastinal dissection technique involving suspension of the esophagus with two traction sutures to facilitate operative access and minimize trauma to adjacent structures. This method significantly reduced operative time without adversely affecting other perioperative outcomes [89].

*Robotic mediastinal lymphadenectomy.* Similar to flexible laparoscopic systems, robotic platforms offer dynamic control of the camera viewing angle, providing a significant advantage during mediastinal dissection by enhancing visualization and surgical precision. Unlike conventional thoracoscopic lymphadenectomy, robotic mediastinal dissection typically begins at the lower mediastinum, followed by lymphadenectomy along the right RLN and subsequently the left RLN [90,91].

In a comparative study evaluating the efficacy of RAMIE vs. MIE, Lei et al. demonstrated the superiority of the robotic approach in achieving extensive mediastinal node dissection. Specifically, RAMIE enabled more meticulous dissection of tracheobronchial (No. 106tbL), paratracheal (No. 106pre), subcarinal (No. 107), left (No. 109L), and right (No. 109R) main bronchial, lower thoracic periesophageal (No. 110), and supradiafragmatic lymph nodes (No. 111) (*p* < 0.05). The study reported a significantly higher number of dissected lymph node stations (*p* < 0.001) and total lymph nodes (*p* < 0.001) with RAMIE without an associated increase in postoperative surgical complications [92]. Thus, RAMIE demonstrated superior thoracic lymphadenectomy performance compared with conventional thoracoscopic approaches, without elevating postoperative morbidity or mortality [93].

*Complete resection of the mesoesophagus.* Drawing inspiration from the principles of mesocolic and mesorectal excision in colorectal cancer, Tachimori et al. introduced the concept of total mesoesophageal resection to enhance anatomical understanding of the mediastinum. In this framework, the esophagus is enveloped by a mesoesophagus composed of lymph nodes, blood vessels, nerves, lymphatic channels, and connective-adipose tissue, enabling a more radical lymphadenectomy [94]. Building on this concept, Lin et al. implemented minimally invasive total mesoesophageal excision in clinical practice, achieving improved local disease control, reduced recurrence rates, and enhanced disease-free and overall survival [95]. Other investigators have adopted these principles to perform extensive mediastinal lymphadenectomies without increasing postoperative morbidity [96,97].

*Concentric three-layered model of the mediastinum.* Fujiwara et al. introduced a concentric anatomical model to enhance understanding of the complex architecture of the superior mediastinum, which is key for esophagectomy with radical mediastinal lymphadenectomy. Centered on the trachea and esophagus, this model delineates fascial planes that separate neural networks, mediastinal vessels, and lymphatic drainage pathways, thereby facilitating anatomical orientation. Its application in both surgical training and clinical practice improves operative precision and enables more radical lymphadenectomies while minimizing injury to vital structures, including the RLN [98].

*Sentinel node mapping and sentinel node navigation.* Although strategies have been proposed to enhance the radicality of esophagectomy, standardized lymph node mapping (Technetium 99 or ICG) has not been incorporated into routine surgical treatment for thoracic EC. The complexity of mediastinal lymphatic anatomy—including multiple collateral pathways and lymph node stations shared with other thoracic organs—continues to challenge efforts to precisely define and execute optimal lymphadenectomy techniques [99].

*Carbon nanotracers.* Qi et al. utilized nanocarbon as a lymphatic tracer during mediastinal dissection to enhance the precision of lymph node station identification. The application of carbon nanotracers has demonstrated improved outcomes in the quality of thoroughness of mediastinal lymphadenectomy [100].

*Functional mediastinal lymphadenectomy.* Some authors have advocated for preservation of the azygos vein arch, demonstrating that mediastinal dissection is feasible at this level through meticulous isolation and controlled manipulation of the vein (Figure 13B,C). Additionally, preservation of the right bronchial artery has been proposed to maintain optimal vascularization of the respiratory tree, particularly in patients who have undergone preoperative chemoradiotherapy. These vascular preservation strategies are encompassed within the concept of functional mediastinal lymphadenectomy, which aims to balance oncologic radicality with preservation of vital structures [101].

### 4.11. Subclass 4B Abdominal Lymph Node Resection

**Figure 14:**
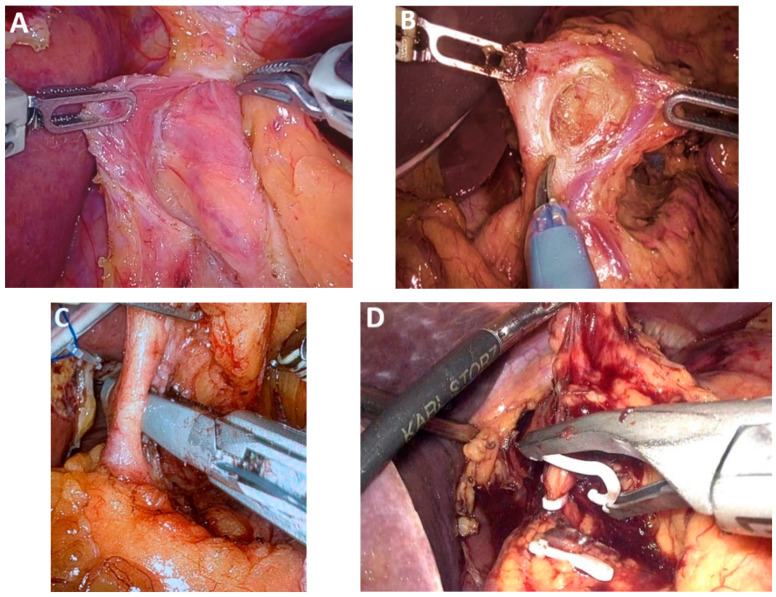
(**A**) Perigastric lymphadenectomy performed at esophagogastric junction. (**B**) Meticulous dissection of the left gastric pedicle. (**C**) Dissection of left gastric artery with vessel sealer. (**D**) Clipping of the left gastric artery with Hem-o-lok clips.

This subclass includes techniques used for perigastric lymphadenectomy. Standard abdominal lymphadenectomy for thoracic esophageal cancer includes lymph node groups 1, 2, 3, 7, 9, 19, and 20 (Japanese Classification of Esophageal Cancer, 12th Edition) [41,42]. Perigastric lymphadenectomy through a minimally invasive approach can be performed laparoscopically, robotically, or hand-assisted (Figure 14A–D).

### 4.12. Subclass 4C Cervical Lymph Node Resection

This subclass includes techniques used for cervical periesophageal lymphadenectomy.

**Figure 15:**
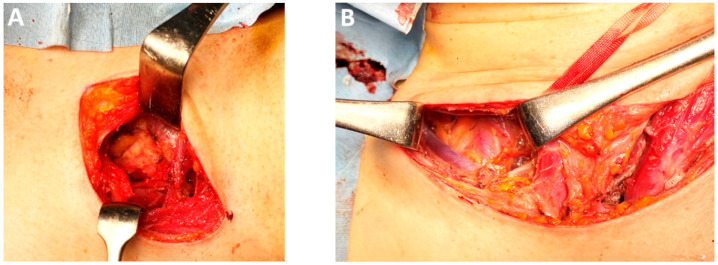

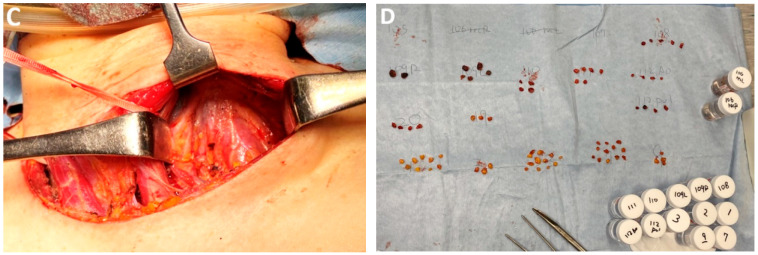
(**A**) Intraoperative view of left cervical paraesophageal node dissection (station No. 101). (**B**) Right prophylactic supraclavicular lymph node dissection involving stations No. 101 and No. 104. (**C**) Left prophylactic supraclavicular lymph node dissection involving stations No. 101 and No. 104. (**D**) Individual dissection of lymph node stations from the esophagectomy specimen enables precise pathological staging and accurate mapping of metastatic spread.

*Left cervical lymphadenectomy.* Unilateral dissection during cervical esophagectomy allows excision of the cervical paraesophageal lymph node (No. 101L) and is facilitated by prior extensive thoracoscopic dissection of the upper thoracic aperture (Figure 15A). This approach enhances anatomical exposure and simplifies esophagus isolation during the neck stage—a technique endorsed by several experts for its precision and efficiency.

*Two-field lymphadenectomy (2FL)/extended two-field lymphadenectomy* vs. *three-field lymphadenectomy (3FL).* The Japanese school of surgery had made significant contributions to EC management, particularly in the 3FL and esophageal reconstruction. 3FL, which includes cervical node stations No. 101L, No. 101R, No. 104L, and No. 104R, enhances staging accuracy by increasing the number of nodes harvested and may reduce local recurrence without significantly elevating postoperative morbidity (Figure 15B–D) [102,103]. MIE with 3FL requires a steep learning curve and is best adopted under the guidance of experienced teams [104]. While some studies associate 3FL with increased morbidity, it offers superior cervical disease control compared with 2FL and extended-2FL [105]. Prophylactic bilateral supraclavicular lymph node dissection remains a selective procedure, recommended only when dictated by tumor location [106].

### 4.13. Class 4—Advances in Esophageal Reconstruction

The fourth class features novel strategies for preparing esophageal substitutes and optimizing graft ascension routes to reduce the incidence of conduit necrosis and anastomotic leakage.

### 4.14. Subclass 4A Esophageal Reconstruction with Stomach

This subclass includes innovations used in the preparation of the future gastric for esophageal reconstruction.

Esophageal reconstruction is as essential as resection, with its success directly influencing postoperative morbidity. The stomach is the preferred conduit due to its robust vascular supply, sufficient length to prevent anastomotic tension, and ease of preparation via laparoscopy. Digestive continuity is typically restored with a single anastomosis [107]. Except in patients with prior gastric resections, the stomach—either whole or tubulized—serves as the standard esophageal substitute.

**Figure 16:**
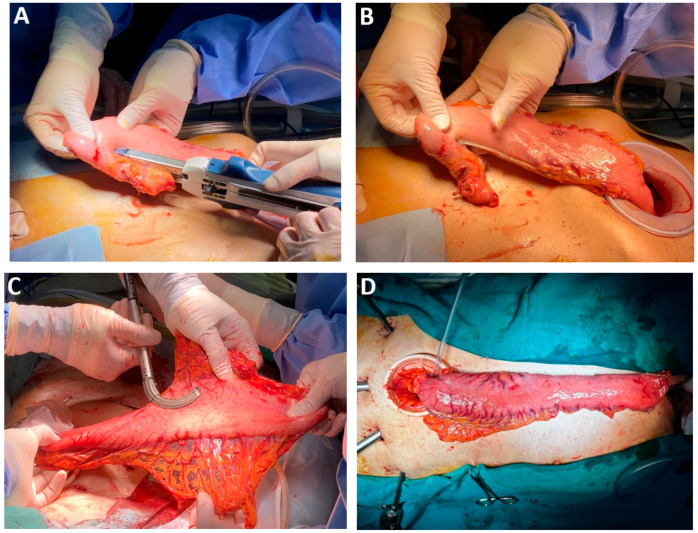
(**A**) Tubulization of gastric conduit using GIA stapler. (**B**) Gastric conduit following tubulization. (**C**) Initiating stomach tubulization using an Endo GIA radial reload. (**D**) Gastric conduit.

The routine tubulization of the stomach remains controversial (Figure 16A–C). Critics argue that dividing the stomach introduced additional risk of graft leakage, particularly at the area where the cartridge staple line overlaps and may compromise perfusion and increase ischemic potential. In patients with prior feeding gastrostomy, gastric graft preparation poses no major technical challenges. During the learning curve, extracorporeal graft preparation via epigastric mini-laparotomy may reduce postoperative complications related to conduit viability (Figure 16D). Intraoperative perfusion assessment using dyes, such as indocyanine green, is recommended to optimize graft viability [108].

In a study by Zhao et al. involving 272 patients undergoing esophagectomy with gastric reconstruction, ICG analysis demonstrated improved outcomes. Patients whose grafts were sectioned 9 cm from the pylorus—preserving the final branch of the right gastric artery—had significantly lower anastomotic leakage rates (*p* = 0.024) and higher blood flow (2.81 cm/a vs. 2.54/s, *p* = 0.001) compared with those with conventional graft preparation at 5 cm from the pylorus. The modified technique also demonstrated superior perfusion and reduced ischemic risk along the staple line (*p* < 0.001), with both groups maintaining a graft width of 3.5 cm [109].

*Ischemic preconditioning of the gastric conduit in the era of MIE.* Ischemic condition of the gastric graft—achieved through partial vascular interruption via embolization or surgical division days to weeks before esophagectomy—aims to enhance perfusion by preconditioning the gastric tissue to reduced blood flow, thereby promoting healing following reconstruction [110].

*Robotically assisted laparoscopic esophageal reconstruction.* Improper use of laparoscopic instruments during gastric graft preparation can result in gastric wall injury, with excessive traction leading to microlesions or microthrombosis that compromise capillary perfusion. Surgical experience plays a pivotal role in minimizing these risks. RAMIE, while offering smoother instrument control, limits the operator to visual feedback alone, potentially increasing the risk of inadvertent trauma. Therefore, a key objective during graft preparation is to preserve tissue integrity and avoid excessive manipulation, particularly from robotic arms [111]. Next-generation robotic platforms equipped with enhanced haptic feedback—such as the da Vinci 5 system with Force Feedback technology (Intuitive, USA)—may reduce the risk of instrumental trauma to the gastric conduit by improving tactile awareness during graft manipulation.

### 4.15. Subclass 4B Esophageal Reconstruction with Colon

This class includes minimally invasive techniques for colon graft reconstruction.

Right or left colon interposition is a life-saving solution for patients with prior gastric surgery or in cases where the gastric conduit becomes ischemic following esophagectomy [112,113].

### 4.16. Subclass 4C Ascension Route

This subclass includes innovations designed to optimize graft ascension to the superior mediastinum or cervical level.

**Figure 17:**
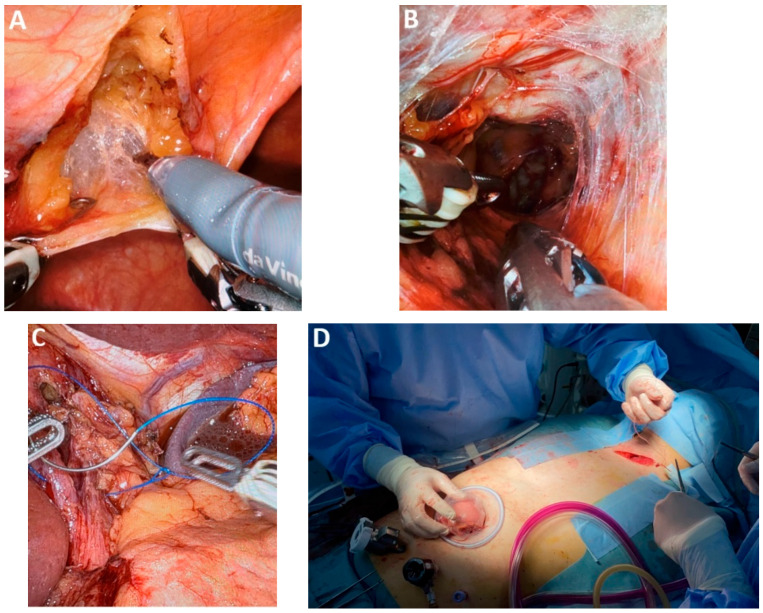
(**A**) Robotic retrosternal dissection using a surgical robot. (**B**) Creation of the retrosternal tunnel for conduit passage. (**C**) Closing the diaphragmatic hiatus. (**D**) Retrosternal ascent of the gastric conduit enclosed within a protective sleeve.

*Retrosternal route* vs. *posterior mediastinal route.* Retrosternal tunneling is readily performed via laparoscopic or robotic approaches (Figure 17A,B). Prior to graft ascent, the diaphragmatic hiatus is routinely closed to prevent transhiatal herniation (Figure 17C) [114]. The retrosternal route minimizes the risk of injury to adjacent organs and provides sufficient length for tension-free anastomosis (Figure 17D) [115]. Although retrosternal gastric graft cancer represents management challenges, resection is feasible through a thoracoscopic approach, avoiding the need for sternotomy [116].

Kikuchi et al. analyzed esophageal reconstruction routes in 9786 patients undergoing esophagectomy, comparing posterior mediastinal (*n* = 3478; 35.5%, including 2986 minimally invasive cases) and retrosternal approaches (*n* = 6308; 64.5%, including 4690 minimally invasive cases). The posterior mediastinal route was associated with lower rates of anastomotic fistula (11.7% vs. 13.8%, *p* < 0.001) and postoperative wound infection (8.4% vs. 14.9%, *p* = 0.005), but a slightly higher incidence of pneumonia (13.7% vs. 12.2%, *p* = 0.040) [117]. Despite these findings, the retrosternal route remains more commonly used in Japan.

In a meta-analysis comparing cervical graft ascension routes, Booka et al. reported a significantly lower anastomotic fistula rate with posterior mediastinal reconstruction compared with the retrosternal approach (*p* < 0.001), with no significant difference in pulmonary complications between the two techniques [118].

*Anterior to the pulmonary hilum route.* Yan et al. demonstrated the feasibility of the anterior pulmonary hilum approach, highlighting its advantage in bypassing the tumor bed and afferent lymphatic pathways—an important consideration for postoperative radiotherapy in locally advanced tumors—without increasing morbidity or mortality [119].

### 4.17. Class 5 Advances in Performing Esophagogastric Anastomosis

This class includes modern techniques used in performing esophagogastric anastomosis during esophageal reconstruction.

### 4.18. Subclass 5A Hand-Sewn Esophagogastric Anastomosis

This subclass covers hand-sewn cervical anastomotic techniques aimed at reducing the incidence of anastomotic fistula—a persistent postoperative complication for which current preventive measures have achieved inconsistent results [120]. While some high-volume EC centers have reported lower fistula rates, a universally effective approach remains elusive and has yet to be adopted globally.

Adequate upper cervicomediastinal dissection is essential for creating sufficient space for esophagogastric anastomosis and minimizing the risk of fistula formation. Graft tension may lead to descent of the anastomosis into the mediastinum, increasing the likelihood of septic and pulmonary complications, particularly when fistula formation involves the right pleura. These technical nuances remain underrepresented in current literature despite their clinical significance.

*Hand-sewn double-layer anastomosis.* He et al. introduced a stepwise technique for cervical esophagogastric anastomosis, beginning with the construction of the posterior wall, followed by suturing the gastric graft to the posterior esophageal wall, and concluding with the anterior layer. This approach contributed to favorable postoperative outcomes, with no reported anastomotic fistulas and low rates of stenosis and acid reflux [121].

### 4.19. Subclass 5B Mechanical Circular Anastomosis

This subclass includes surgical techniques that use circular staplers in performing esophago-gastric anastomosis.

**Figure 18:**
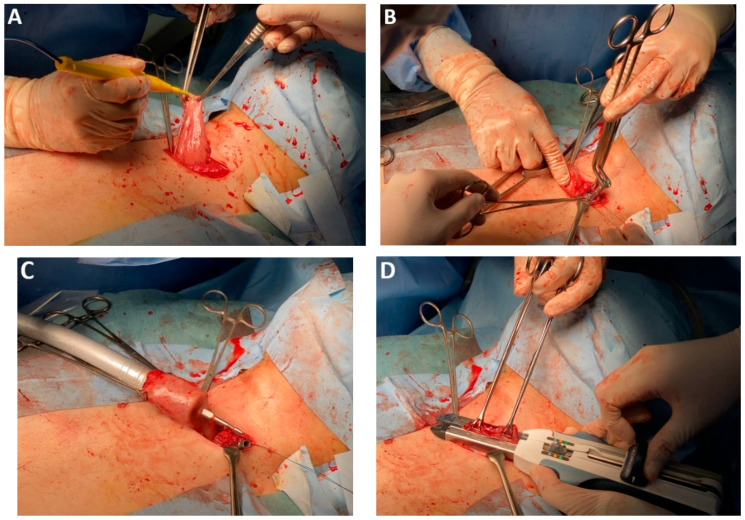
(**A**) Intraoperative view showing preparation of gastric graft for cervical anastomosis. (**B**) Placement of envil into the esophageal stump. (**C**) End-to-side mechanical esophago-gastric anastomosis performed using a circular stapler. (**D**) Closure of gastric conduit.

*Mechanical circular anastomosis*. Circular mechanical *end-to-side* anastomosis performed using a 25 mm stapler remains one of the most widely utilized techniques in digestive surgery (Figure 18A–D). Its popularity is attributed to the preservation of vascular integrity at the anastomotic margins and the relative ease of execution, both of which contribute to favorable surgical outcomes [122,123].

*Hybrid stapled method.* Fujimoto et al. employed a hybrid stapling technique for cervical esophagogastric anastomosis, initiating with a 21 mm circular stapler to create an end-to-side anastomosis between the cervical esophagus and posterior gastric wall. To widen the anastomotic lumen, a 30 mm linear stapler was applied over the circular anastomosis, followed by a 15 mm side-to-side extension. The entry site was then closed using a linear stapler. This approach was associated with a reduced incidence of anastomotic stenosis [124].

*Robotic hand-sewn anastomosis.* The feasibility of robotically performed intrathoracic hand-sewn esophagogastric anastomosis has been established. Ny Ayl et al. reported outcomes comparable to those of mechanical anastomosis, with similar rates of anastomotic fistula and stenosis [125]. To enhance surgical training, Wong et al. incorporated a simulation model for robotic esophagogastric anastomosis into educational programs focused on esophageal and esophagogastric junction surgery [126].

*Supercharged microvascular cervical anastomosis.* Among the various factors influencing cervical esophagogastric anastomotic healing, insufficient vascular supply remains one of the most frequently implicated. In a comparative study, Takeda et al. reported a 26.5% increase in perfusion following venous microanastomosis. Although the rate of anastomotic complications dropped to zero, the clinical outcomes did not reach statistical significance. A reduction in anastomotic stenosis was also observed. Given its technical complexity, this approach may be reserved for selected patients [127].

### 4.20. Subclass 5C Mechanical Linear Anastomosis

**Figure 19:**
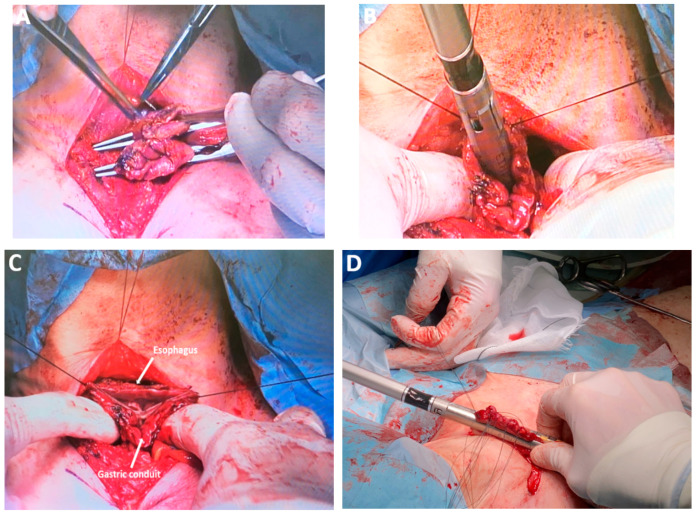
(**A**) First step in performing a side-to-side cervical eso-gastric mechanical anastomosis with a linear Endo GIA stapler. (**B**) Linear stapler firing. (**C**) Intraoperative view following the stapler firing. (**D**) Final closure of the cervical anastomosis.

This subclass includes surgical techniques employing linear staplers for esophagogastric anastomosis. In a comparative analysis of mechanical versus manual anastomoses, Davey et al. reported a significant reduction in anastomotic fistula rates with stapler use. However, circular staplers were associated with a higher incidence of anastomosis stenosis. Although technically more demanding, linear stapling appears to offer superior outcomes in anastomotic healing with respect to both fistula formation and stenosis (Figure 19A–D) [128].

## 5. Discussion

Modern surgical management of EC prioritizes minimally invasive techniques, aiming to optimize oncologic outcomes while minimizing perioperative morbidity. Planning for esophageal resection cannot be performed without a detailed imaging evaluation, which may include 3D reconstruction of the mediastinal anatomy. MIE allows the simplification of postoperative healthcare, the reduction of the incidence of postoperative complications, especially pulmonary, the reduction of hospitalization time, and a faster social reintegration. Contemporary approaches incorporate comprehensive lymphadenectomy strategies that enable adequate nodal clearance without compromising postoperative safety. The adoption of MIE has introduced technical challenges, particularly in replicating the exposure and maneuverability of open procedures. Nonetheless, over the past two decades, MIE has evolved into the preferred approach for EC resection, supported by accumulating evidence of its safety, efficacy, and favorable postoperative recovery profile [129]. In a 2022 international multicenter study, de Groot et al. reported that totally minimally invasive esophagectomy was significantly more prevalent in high-volume centers, accounting for 53% of esophagectomy procedures, compared with low-volume institutions (*p* = 0.002) [130].

Three key domains have emerged as focal points in the evaluation of MIE for EC: reduction in pulmonary complications, mitigation of anastomotic leak rates, and the attainment of oncologic outcomes comparable to or exceeding those achieved with open esophagectomy. Multidisciplinary teams have increasingly investigated the impact of minimally invasive techniques across these parameters.

Textbook criteria for minimally invasive esophagectomy establish benchmarks that are difficult to achieve consistently in routine surgical practice. In a cohort of 236 patients, Seika et al. demonstrated that robotic-assisted MIE was associated with significantly improved postoperative outcomes compared with conventional MIE when performed under ideal conditions [131]. The implementations of postoperative quality indicators should be contextualized by the extent of surgical resection in patients previously treated with neoadjuvant chemoradiotherapy. Bolger et al. proposed a threshold of 15 lymph nodes resected as an objective for improved survival, a metric that may also be used to evaluate surgical performance [132].

To meet the increasingly stringent surgical quality indicators, continuous efforts are directed toward advancing the technological components of surgical care. In the field of minimally invasive esophageal cancer surgery, innovations have emerged both through rapid development and by translational adoption from other surgical domains.

The Classification of Innovations in Minimally Invasive Esophagectomy seeks to clarify the evolving landscape of this complex procedure and support clinical decision-making. By promoting a structured understanding and integration of each innovation into current practice, this approach aims to enhance surgical outcomes in EC treatment.

Among the various innovative elements introduced, particular emphasis should be placed on those that demonstrably influence the conduct of the surgical procedure and significantly improve postoperative outcomes.

The first category of technological advances comprises innovations in technical components that optimize surgical conditions in the operative field. Enhanced intraoperative imaging facilitates the identification of fine anatomical landmarks and enables safe dissection of vital mediastinal structures. As a result, the number of lymph nodes retrieved in MIE specimens is consistently adequate, reflecting improved visualization and precision during lymphadenectomy [133].

The use of instruments specifically adapted for both thoracoscopic and laparoscopic stages of MIE is indispensable to ensure procedural safety and reduce operative time [134,135]. Although MIE entails substantial costs related to endoscopic surgical equipment, the selection of the surgical approach should be guided by patient benefit rather than economic constraints [136,137].

Recent technological advances allow the use of artificial intelligence in the treatment of thoracic esophageal cancer. AI is already implemented and used on endoscopic platforms to identify early EC [138]. Virtual reality support can thus be useful in reducing postoperative complications, especially recurrent nerve injury [139]. An important contribution of virtual reality is found in the learning process of performing esophagectomy [140]. Machine learning was used to assess the gastric conduit perfusion in an experimental model for MIE [141]. Artificial intelligence can also be used to explain complex procedures, such as minimally invasive esophagectomy, to patients with EC.

The use of an endobronchial blocker represents a key anesthetic-surgical element influencing the thoracoscopic stage of MIE. This technique facilitates tracheal mobilizations, thereby improving access to the left recurrent laryngeal nerve while minimizing the risk of tracheal membrane injury compared with conventional Carlens selective intubation [142].

The second category of innovations focuses on optimizing the surgical approach to facilitate esophageal dissection within the mediastinum while minimizing operative trauma. One technical refinement with notable impact on both the duration and conditions of MIE is the transection of the upper thoracic esophagus, which significantly improves access to the superior mediastinum, particularly in the region of the recurrent laryngeal nerves. In cases involving challenging dissection of mid-thoracic esophageal tumors with close adherence to adjacent thoracic structures, lower thoracic esophageal transection may be employed to enable bidirectional dissection and improve operative control.

Nonetheless, the decision to pursue a total MIE should not be driven solely by the intent to minimize surgical trauma. Rather, it must be grounded in intraoperative anatomical realities, which may necessitate conversion to a hybrid approach in select cases [143]. Intraoperative findings also can lead to changes in surgical tactics during the course of esophagectomy, including sometimes conversion to open surgery if necessary, which is why it is important that all recent imaging of the patient be reviewed at an interval as close as possible to the operation. Regarding hybrid esophagectomy, it is considered an excellent transition from the open approach to totally MIE. Using a hybrid approach, the surgical team can choose whether the laparoscopic or thoracoscopic stage becomes a priority in the learning curve, thus reducing this learning curve [144,145].

Lin et al. proposed a modification to the operative sequence of minimally invasive esophagectomy, initiating with laparoscopic gastric mobilization, retrosternal ascent, and cervical anastomosis, followed by thoracoscopic mediastinal dissection with the esophagus transected at both ends. This approach (reverse esophagectomy) facilitates mediastinal dissection and lymphadenectomy, particularly benefiting early-career esophageal surgeons. This technique was associated with reduced operative duration, notably during the thoracic phase, and demonstrated procedural feasibility [146]. This technique is reproducible, provided that the resectability of the esophageal tumor is confirmed preoperatively with high certainty. Accurate staging and multidisciplinary evaluation are essential to ensure appropriate patient selection and to avoid intraoperative challenges related to unexpected tumor extension. Considering these aspects, upper thoracic esophageal tumors may present intraoperative challenges due to their proximity to the tracheal membrane. Despite thorough preoperative staging and assessment, unexpected adhesions or invasion can complicate dissection and compromise airway safety.

EC is commonly diagnosed in elderly patients with multiple comorbidities, contributing to a high incidence of post-esophagectomy complications [147,148]. Consequently, minimizing surgical invasiveness is fundamental, reinforcing the need to leverage all available resources toward this goal. In an international study, Riccio et al. evaluated the minimally invasive transcervical approach and suggested its potential as a viable alternative, particularly for patients at elevated risk of thoracic complications. MICE remains under standardization and requires further validation to establish its safety and efficacy [149]. In a learning curve analysis, Hu et al. reported that proficiency in the transcervical technique was achieved after 27 cases for the primary surgeon and 35 cases for the assistant surgeon, as reflected by reduced operative times [150].

Technological advancements classified as level three aim to enhance the oncologic radicality of MIE without increasing postoperative morbidity. Nonetheless, the fundamental goal of surgical intervention remains to complete resection of esophageal tumors and associated lymphatic drainage territories, adhering strictly to oncologic principles and resection margins.

Current perfusion assessment techniques for esophageal reconstruction primarily focus on quantifying blood flow and tissue oxygenation at the anastomotic site to minimize fistula risk. However, limited attention has been given to the comprehensive vascular profile of the gastric graft, including arterial inflow, venous outflow, and lymphatic drainage. These components play a central role in graft viability and healing. Further investigation into the integrated vascular dynamics of the conduit is warranted, as existing evaluations remain insufficient and underexplored. Beyond technical refinements in esophageal reconstruction using gastric conduits, intraoperative assessment of graft perfusion enables stratification of patients at high risk of anastomotic fistula or graft necrosis. For these high-risk individuals, targeted postoperative monitoring and infection control strategies may be warranted [151]. Although impaired graft vascularization remains a leading contributor to anastomotic failure, additional coexisting risk factors must be considered when evaluating anastomotic integrity [152].

A notable advancement involves the creation of a retrosternal tunnel via laparoscopic or robotic techniques, allowing cervical graft ascent while positioning the conduit outside the pleural cavity—an approach that may enhance pulmonary recovery.

Innovations in esophagogastroanastomosis, classified at level five on the surgical innovations scale, primarily aim to reduce anastomotic fistula rates. However, the adoption of MIE has not translated into a decreased risk of anastomotic leakage. While MIE offers clear postoperative benefits over open esophagectomy—solidifying its role as the current standard—its impact on anastomotic healing has been less favorable. In a cohort analysis of 10,607 esophagectomies, Khaitan et al. reported increased utilization of conventional and robotic-assisted minimally invasive techniques, yet a persistently higher incidence of fistula-related complications remained among patients undergoing MIE [153].

The execution of esophagogastric anastomosis has been significantly enhanced by the integration of advanced stapling technologies, particularly the use of linear and circular staplers. Cervical esophagogastric anastomosis continues to be performed hand-sewn worldwide, reflecting both the technical complexity of the procedure and the limited applicability of mechanical devices in this anatomical region. In a randomized clinical trial, intrathoracic anastomosis was associated with a lower incidence of anastomotic fistula and severe complications compared with cervical anastomosis, albeit at higher procedural costs. Despite its clinical advantages, intrathoracic anastomosis remains technically demanding via minimally invasive approaches, necessitating specialized training in high-volume centers to ease the learning curve. Importantly, a fistula at the intrathoracic site may result in life-threatening complications, whereas cervical anastomotic fistulas are typically amenable to conservative management in most cases [154].

Procedural standardization and subspecialization in esophageal cancer surgery have been associated with marked improvements in postoperative outcomes. Notably, the incidence of anastomotic leakage decreased from 14% to 1.6% following implementation of ultraspecialized care (*p* < 0.0001) [49]. Standardization of surgical techniques in EC management remains a central objective of patient-centered treatment guidelines, aiming to ensure reproducible outcomes. While its advantages—reduced operative time, lower postoperative morbidity, and enhanced team efficiency—are well recognized, implementation is hindered by heterogeneity in available technologies and variability in surgical training, often shaped by mentor-specific esophagectomy approaches [155,156].

Despite significant technological progress in the minimally invasive management of EC, several resources and innovations remain underutilized, offering further potential for refinement and improved outcomes.

Recognizing that surgical intervention alone does not encompass the full spectrum of therapeutic solutions, complementary efforts are underway to enhance treatment efficacy and survival in EC. These include integrations of immunochemotherapy protocols and emerging research into the esophageal microbiome, both of which hold promise for refining patient stratification and optimizing multimodal management.

Given that many novel surgical technologies and instruments are not yet widely adopted, it is essential to promote their dissemination and evaluate their feasibility across high-volume experienced centers. Broadening the use of such innovations not only supports industry stakeholders but also fosters continued surgical advancement, ultimately translating into improved patient outcomes. This study has several limitations inherent to its narrative style, which should be considered when interpreting the results.

## 6. Conclusions

The surgical management of the EC remains a complex endeavor, typically reserved for high-volume centers with specialized expertise, due to its association with significant morbidity, mortality, and prolonged postoperative recovery.

MIE has emerged as a less traumatic alternative, offering improved postoperative outcomes. MIE facilitates streamlined postoperative care, reduces the incidence of complications, particularly pulmonary, and shortens hospital stays, thereby accelerating patient recovery and faster social reintegration. The integration of minimally invasive techniques has markedly advanced esophageal oncologic surgery, enhancing procedural safety, oncological radicality, and functional preservation. The improvements have translated into a better postoperative quality of life for patients.

Recent technological innovations, including the incorporation of artificial intelligence into laparoscopic and robotic platforms, hold promise for further refinement of esophageal cancer surgery. The active involvement of esophageal surgeons in the development of these technologies ensures their practical applicability and accelerates their adoption in clinical practice. Such advancements can simplify and secure technically demanding stages of esophagectomy.

Establishing a structured classification of technological and procedural innovations in the minimally invasive treatment of esophageal cancer may provide a valuable reference for future generations of esophageal surgeons in this continuously evolving field.

## Figures and Tables

**Table 1:** Classification of innovations in minimally invasive treatment of thoracic esophageal cancer.

Classification of Innovations	Subclasses	Definition	Objectives
1—Technological innovations	1a—camera technology 1b—surgical instruments 1c—auxiliary equipment	Improvements and innovations in camera, Development of new instruments and other auxiliary equipment, including anesthetic measures	Optimization of intraoperative visualization to enhance surgical precision and reduce operative time Integration of ergonomic and functionally advanced surgical instruments to facilitate dissection and improve surgeon efficiency Deployment of surgical and anesthetic equipment designed to enhance procedural safety and minimize intraoperative and postoperative complications
2—Advances in surgical approach	2a—transthoracic MIE 2b—transthoracic RAMIE 2c—transcervical MICE	Improvements in surgical approach Optimization of surgical technique Improvements of MIE, RAMIE and MICE	Reducing surgical trauma Improved access to difficult anatomical areas Reduced risk of intraoperative incidents and accidents Shorter learning curve
3—Advances in lymph nodes dissection	3a—thoracic 3b—abdominal 3c—cervical	New modalities of performing lymph node dissection	Effectiveness of operating stages facilitating radical lymphadenectomy Reducing complications associated with lymphadenectomy
4—Advances in graft preparation	4a—stomach 4b—colon 4c—ascension route	New modalities to prepare esophageal substitute New ascension routes	Minimizing the occurrence of graft necrosis
5—Advances in performing esophagogastric anastomosis	5a—hand-sewn 5b—mechanical circular 5c—mechanical linear	New techniques for performing cervical or intrathoracic anastomoses	Reducing the risk of anastomotic leakage

## Data Availability

No new data were created or analyzed in this study. Data sharing is not applicable to this article.

## Abbreviations

The following abbreviations are used in this manuscript:

MIE	Minimally invasive esophagectomy
RAMIE	Robotic-assisted minimally invasive esophagectomy
MICE	Minimally invasive transcervical esophagectomy
EC	Esophageal cancer
RLN	Recurrent laryngeal nerves
ICG	Indocyanine green
ICG-TBF	Tracheobronchial fluorescence
AI	Artificial intelligence
TD	Thoracic duct
HSI	Hyperspectral imaging
OXEI	Oxygen saturation endoscopic imaging
RATME Robot-	Assisted Transcervical Esophagectomy
2FL	Two-field lymphadenectomy
3FL	Three-field Lymphadenectomy
